# Saccharification of pretreated sawdust by *Aspergillus niger* cellulase

**DOI:** 10.1007/s13205-015-0284-7

**Published:** 2015-03-19

**Authors:** A. Sridevi, G. Narasimha, G. Ramanjaneyulu, K. Dileepkumar, B. Rajasekhar Reddy, P. Suvarnalatha Devi

**Affiliations:** 1 Department of Applied Microbiology, Sri Padmavathi Mahila University, Tirupati, AP India; 2 Department of Virology, Sri Venkateswara University, Tirupati, AP India; 3 Department of Microbiology, Sri Krishnadevaraya University, Anantapuramu, 515 003 AP India

**Keywords:** *Aspergillus niger*, Lignocelluloses, Pretreatment, Sawdust, Cellulases

## Abstract

The efficiency of two methods of pretreatment (NaOH and H_2_O_2_) on lignocelluloses—saw dust, wheat straw, sugarcane bagasse and rice bran—was compared in the present study. Alkali treatment of lignocelluloses relatively removed more hemicelluloses and lignin leaving behind cellulose content in the residues than peroxide treatment. Crude cellulase of *Aspergillus niger*, produced on the pretreated sawdust with highest cellulose content, was further tested for the release of soluble and reducing sugars during the saccharification process of same pretreated saw dust. The saccharification process of the pretreated sawdust with enzyme was optimized for pH, temperature, and substrate concentration and proceeded optimally at pH of 5.0, 50 °C and 0.5 % pretreated sawdust. The rate of saccharification with crude enzyme of *A. niger* on alkali-treated sawdust was found to be maximum (23 %) as against 5.4 % on native sawdust under optimal conditions after 48 h. The present study indicates NaOH-treated sawdust as a potential raw material for both production of cellulase and saccharification in a large scale.

## Introduction

Lignocellulose is the major structural component of plant biomass such as woody and non-woody plants and represents major part of renewable organic matter and substrate available for conversion to fuels. On a worldwide basis, terrestrial plants produce 2.3 × 10^11^ metric tons/year (dry weight basis) of wood, which is equivalent to 7 × 10^10^ metric tons of coal or about two-thirds of the world’s energy requirement. Available cellulosic feed stocks from agriculture and other sources are about 180 million tons/year (Lynd et al. 2003). Furthermore, tremendous amounts of cellulose are available as municipal and industrial wastes which today contribute to our pollution problems. Thus, there is great interest in the use of cellulosic biomass as a renewable source of energy via breakdown to sugars that can then be converted to liquid fuel by cellulases.

Cellulase is a multi-enzyme complex involving the synergistic action of exo-β-1, 4 glucanase (3.2.1.91), endo- β-1, 4 glucanase (3.2.1.4) and β-glucosidase (3.2.1.21). Cellulolytic enzymes can be produced upon growth of cellulolytic organisms on the cheap and best substrates like lignocelluloses. A variety of lignocellulosic substrates tried for industrial production of cellulase enzymes in submerged fermentation and solid state fermentation included sugarcane bagasse, wheat bran, rice bran, gram bran, wheat straw, rice straw, rice husk, soy-hull, sago hampas, grape vine, trimming saw dust, corn cobs, coconut, coir pith, banana waste, tea waste, cassava waste, palm oil mill waste, aspen pulp, sugar beet pulp, sweet sorghum pulp, apple pomace, peanut meal, rapeseed cake, coconut oil cake, mustard oil cake, cassava flour, wheat flour, corn flour, steamed rice and stream pretreated willow, depending on availability of substrates in different regions (Kumar et al. 2009; Zheng et al. 2014; Zheng and Rehmann 2014; Anwar et al. 2014). Sridevi et al. (2009) demonstrated high production of cellulase by a local isolate *Aspergillus niger* on commercial cellulose and natural lignocelluloses. Thus cellulase enzyme produced on cheap raw materials—lignocelluloses—in this fashion can be used for conversion of lignocelluloses into soluble sugars in a saccharification process which is a platform for potential development of technologies for production of fuels, chemicals, etc. However, use of natural lignocelluloses in enzymatic saccharification generated low yields of fermentable sugars because of less inaccessibility of cellulose component in lignocellulose to enzyme attack due to encasement of cellulose component in a matrix of lignin and hemicellulose.

Pretreatment is necessary to reduce recalcitrance of lignocellulosic biomass for enhancing cellulose component to enzymatic hydrolysis in subsequent steps. Even though a number of pretreatment methods have been tried individually or in combination to loosen structure of lignocellulosic biomass like milling, grinding, pyrolysis, high-energy radiation, alkaline or acid hydrolysis, gas treatment, hydrogen peroxide treatment, organic solvent treatment, wet oxidation and biological treatment, etc. (Kumar et al. 2009; Chaturvedi and Verma 2013; Muhammad Irfan et al. 2014; Marzieh Badiei et al. 2014; Langan et al. 2014), alkaline and acid pretreatment methods are popular (Iroba et al. 2013; Chen et al. 2013). Acid-pretreated methods were quite effective at only high temperature on different lignocellulosic biomasses with high recovery of cellulose compounds due to removal of hemicelluloses (Sun et al. 2014; Kapu and Trajano 2014). But, the most drawbacks with acid pretreatment methods were formation of inhibitor—furfural and hydroxyl methyl furfural which may affect fermentation in subsequent stages. Alkaline pretreatment methods facilitate removal of lignin component from lignocellulosic biomass leaving behind polysaccharide compound (Chen et al. 2013). In view of retaining of polysaccharide component in lignocellulosic biomass and operation of alkaline pretreatment method at low temperature (less energy process) and little information on influence of peroxide under alkaline conditions on structure of lignocellulosic biomass, the present study has been focussed on comparison of two methods—alkali method and alkaline peroxide method—for treatment of lignocellulose and understanding of enzymatic saccharification of pretreated lignocellulose.

## Materials and methods

### Production of cellulase

High cellulase enzyme production by a local fungal isolate of *A. niger* on the medium with following ingredients in g/L: NaNO_3_—1.0; K_2_HPO_4_—1.0; MgSO_4_·7H_2_O—0.5; KCl—0.5; FeSO_4_·7 H_2_O—0.01; pretreated sawdust—1.0 in a liter of distilled water with pH of 5.0 in submerged fermentation was demonstrated in our earlier study (Sridevi et al. 2009). The same medium was used for production of cellulase by the same local isolate of *A. niger* in the present study. Spores of *A. niger* (Narasimha et al. 1999) were produced on potato dextrose agar slants after 7 days of growth at 30 °C. Three milliliters of spore suspension of *A. niger* with density of 2 × 10^8^ spores/ml was prepared from slants with sterile distilled water and was inoculated aseptically into enzyme production medium. The submerged culture was incubated at 30 °C on a rotary shaker (180 rpm) under axenic conditions. The filtrate, obtained after removal of mycelial mat by filtration through Whatman filter paper No.1 and followed by centrifugation at 10,000*g* for 10 min, was used as a crude enzyme source. Activity of different components in the crude enzyme source was measured in terms of Fpase, CMCase and β—glucosidase.

### Enzyme assays

#### Filter paper assay

Filter paper activity indicates total cellulolytic activity resulting from combined action of different enzyme components present in the culture filtrate. Filter paper activity of the culture filtrate of *A. niger* was determined according to the method of Mandels and Weber (1969). Whatman filter paper strips (50 mg) in one ml of 0.05 M sodium citrate buffer, pH 4.8, at 50 °C with 1 ml of enzyme source was incubated for 60 min at 50 °C. The reducing sugars produced during incubation were determined by di-nitro salicylic acid (DNS) method (Miller 1959). Activity of cellulase was expressed in filter paper units (FPU). One unit of filter paper unit was defined as the amount of enzyme releasing 1 µmol/min of reducing sugar from filter paper.

#### Carboxymethyl cellulase assay

Activity of endoglucanase in the culture filtrate was determined by carboxymethyl cellulase method (Ghosh 1987). Assay of endoglucanase (Carboxymethyl cellulase) involved incubation of 1.0 ml of 1 % carboxymethyl cellulose (Merck) in 0.2 M acetate buffer (pH 5.0) with 1 ml of enzyme solution at 50 °C for one hour in a water bath. The reducing sugar formed in the reaction mixture was determined by di-nitro salicylic acid (DNS) method (Miller 1959). One unit of endoglucanase activity was defined as the amount of enzyme releasing 1 µmol/min of reducing sugar.

#### β-D-glucosidase assay

Titre of β-glucosidase present in the culture filtrate was based on the method of Herr (1979). For the determination of β-D-glucosidase activity, the assay mixture with 0.2 ml of 5 mM *p*-nitrophenyl β-D-glucopyranoside (PNPG, Merck) in 0.05 M citrate buffer pH 4.8 and 0.2 ml of culture filtrate was incubated for 30 min at 50 °C and the reaction was halted by adding 4 ml of 0.05 M NaOH-glycine buffer (pH-10.6). The yellow-coloured *p*-nitrophenol liberated was determined in a Spectrophotometer (ELICO-SL 164) at 420 nm. One unit of β-glucosidase activity was defined as the amount of enzyme liberating 1 µmol/min of *p*-nitrophenol under standard assay conditions.

### Pretreatment of lignocelluloses

Different lignocelluloses—saw dust, wheat straw, sugarcane bagasse and rice bran—were collected locally, chopped into 1–2 cm lengths and were treated separately by two pretreatment processes.

### Alkaline pretreatment

Dried lignocellulose samples were incubated in 1 N NaOH solution at the rate of 50 ml/g at room temperature for 24 h following which the slurries were washed repeatedly with water to a neutral pH, and then oven dried at 60 °C to a constant weight.

### Oxidative pretreatment

Samples (one gram) of lignocellulose of each type were placed in 50 ml of distilled water containing 1 % H_2_O_2_ (W/V aqueous solution). The suspension was adjusted to pH 11.5 with NaOH and stirred gently at room temperature (25 °C) for 20 h. The insoluble residue was collected by filtration, washed with distilled water until neutrality and dried at 110 °C overnight (Gould 1984). The oven-dried materials were packed in polythene bags and stored under dry conditions at room temperature until use.

### Gravimetric analysis

The different components present in lignocelluloses used in this study were determined by sequential chemical extraction according to the method of Harper and Lynch (1981). One gram sample of each pretreated lignocellulose and native lignocellulose (untreated sample dried at 60 °C for 16 h) were weighed into 125 ml conical flasks.

For the determination of hot water-soluble fraction, 75 ml of distilled water was added to 1 g of native and pretreated lignocelluloses and boiled gently for 1 h. The water was changed and the mixture was boiled again for 1 h. The residue was recovered and washed with cold water and dried at 60 °C for overnight and carefully weighed. Loss in weight of residue after hot water treatment was hot water-soluble fraction.

To determine hot ethanol soluble fraction, 100 ml of ethanol was added to the solid sample obtained at the end of the previous treatment and boiled for 2 h. The residue obtained from each lignocellulose was washed twice with ethanol and twice with water, dried at 60 °C. Weight difference in solid residue recovered at the end of water process and at the end of ethanol process was hot ethanol fraction.

To find out the lignin content in all lignocellulosic samples with/without treatment used in this study, 30 ml of water, 2 ml of 10 % (v/v) aqueous acetic acid solution and 0.6 g of sodium hypochlorite were added to the residues obtained at the end of hot ethanol process. The mixture was heated at 70 °C for 1 h and then 2 ml of acid and 0.6 g of sodium hypochlorite were added again. After 2 h of treatment, the residues were washed five times with water, twice with acetone and once with ether, dried then at 105 °C for 90 min and weighed. The weight difference between residue recovered after ethanol treatment and residue recovered after the present treatment gives the lignin content.

To measure the hemicellulose content, 20 ml of 24 % KOH solution was added to the solid residue recovered after the determination of lignin content and left then to stand for 2 h at 20 °C. The residues were then washed five times with water followed by one washing with 5 % aqueous acetic acid solution, one washing of water, one washing of acetone and one washing of ether. Finally, the samples were dried at 60 °C for 90 min and weighed. The weight difference gives the hemicellulose content. The weight of the remaining residue after removal of lignin and hemicellulose was taken as the cellulose content.

### Enzymatic sachaarification of native and alkali treated sawdust

Only the lignocellulose—sawdust with high cellulose—was used for the study of saccharification. Sample of 2.5 g (W/V) each from native and alkali-treated substrate was placed in 50 ml of 0.05 M citrate buffer in 250 ml stoppered conical flasks. Prior to addition of the enzyme preparation to the native and pretreated sawdust samples, the substrate and buffer mixture were autoclaved for 20 min at 121 °C to prevent contamination. Three milliliters of *A. niger* crude culture filtrate (FPU—6.3 U/ml, CMCase—7.2 U/ml, β-Glucosidase—0.30 U/ml) were added to the above flasks and incubated at 180 rpm at 50 °C with pH of 5.0 for 72 h. Flasks in which no enzyme was added served as control. Samples from each set were taken after 24, 48 and 72 h. The reaction was stopped by keeping tubes on ice cold water bath and the contents of the tubes were centrifuged for 10 min at 4 °C. The reducing sugar content in the supernatant was measured by DNS method (Miller 1959). The percent hydrolysis was calculated using the following equation:

$${\text{Percent of hydrolysis}} = \frac{{{\text{Total sugars}} ({\text{g}}) \times \;0.9 \times 100}}{{{\text{Weight of alkali}}\,\mathcal{ - }\,{\text{treated saw}}\;{\text{dust}} ({\text{g}})/ {\text{untreated saw dust}}}}$$

In order to find out influence of temperature on saccharification process, another set of flasks with the same reaction mixture were incubated at different temperatures—30, 40. 50 and 60 °C. In another experiment, saccharification was carried out with incubation of the same reaction mixture in the medium buffered with 0.05 M citrate buffer adjusted to different pH—4.5, 5.0, 5.5 and 6.0. In another experiment, to study the influence of substrate concentration on enzymatic hydrolysis, four different concentrations of both native and treated sawdust (0.5, 1.0, 2.5 and 5.0 %) were included in 50 ml of 0.05 M citrate buffer and incubated after addition of crude enzyme at pH 5.0 and 50 °C with suitable controls. In all these experiments, the reaction was arrested and flasks were processed in the same fashion as mentioned earlier.

### Statistical analysis

All experiments were performed in triplicate and the related data were expressed as mean ± standard deviation. The experimental data from enzymatic saccharification were statistically analysed using SPSS program by one-way analysis of variance (ANOVA) followed by Duncan’s multiple range method test to separate the means. Differences in means were judged significant when *p* values for the null hypothesis were 0.05 or less (Estrada et al. 1988).

## Results and discussion

Analysis of composition of lignocellulosic masses used in the present study indicated that the native and untreated lignocelluloses—sawdust, sugarcane bagasse, wheat straw and rice bran—contained cellulose component to the extent of 47.7, 43.7, 43.9 and 45.7 %, respectively (Table 1). Hemicellulose is the second dominant component (16.9, 15.8, 15.0 and 14.0 %) in the same four native lignocelluloses, while lignin constituted 12.4, 12, 10.9 and 11.8 % of total dry weight of raw lignocelluloses, respectively (Table 1). Results of the present study on composition of hardwood such as saw dust and rice straw in terms of cellulose, hemicellulose and lignin were comparable to the results of other studies on composition of the same lignocellulosic masses reported earlier (Hu 2006; Shulga et al. 2007; Song et al. 2012; Amiri et al. 2014). However, cellulose content of wheat straw and sugarcane bagasse in the present study was on the higher side in comparison to other studies (da Silva et al. 2010; Rabelo et al. 2011; Yang et al. 2011a, b; Wang et al. 2014; Hui et al. 2014). Variation in composition of lignocellulosic masses in different studies may be attributed to different agronomic and cultural practices adopted for growth of the plants and different methods employed for analysis of composition. Two pretreatment methods—alkali method and alkaline peroxide method—were performed on four lignocelluloses such as sawdust, sugarcane bagasse, wheat straw and rice bran at low (ambient) temperature in this study. Alkali treatment with NaOH at room temperature resulted in a considerable removal of different components, resulting in an increase in cellulose content from 47.7 to 63.1 % in pretreated sawdust, 43.7–57.3 % in wheat straw, 43.9–56.1 % in sugarcane bagasse and 45.7–53.6 % in case of rice bran (Table 1). The treatment of alkaline hydrogen peroxide increased cellulose content from 47.7 to 50 % in sawdust, 43.7–55.0 % in wheat straw, 43.9–51.6 % in sugarcane bagasse and 45.7–52.5 % in rice bran. Lignocelluloses treated by both methods were referred to as partially delignified lignocelluloses. Alkali-treated sawdust had retained more cellulose than other lignocelluloses in the present study. Use of 2.4 % NaOH at lower temperature −80 °C for 70 min caused maximum removal of 43 % of lignin (Uzunlu et al. 2014) in poppy stalks. The results thus obtained in the present study are in agreement with Thakur et al. (2013) who also reported dilute NaOH pretreatment as an effective method. Similarly, pretreatment of substrates with alkali may result in the swelling of the particle causing easy lignin removal and cellulose depolymerization (Damisa et al. 2008). But final content of cellulose in lignocelluloses after treatment with oxidative treatment was lower than that yielded from lignocelluloses after treatment with alkali method. With increasing concentration of H_2_O_2_ up to 5 % under alkaline conditions, greater destruction of all cell wall components in lignocelluloses with concomitant decline in cellulose content and weight loss was observed (Irfan et al. 2011). Similarly, rather higher oxidation of cellulose component in comparison to other components in lignocellulose could be a reason for low recovery of cellulose with alkaline peroxide method in the present study. On the other hand, increasing the amount of alkaline hydrogen peroxide up to 4.3 % in the pretreatment resulted in progressive reduction of lignin with concomitant increase of cellulose in cashew apple bagasse (CAB) and lignin was reduced from original composition of 36–7 % in the pretreated solid residue (Correia et al. 2013). Among five different pretreatment methods employed on sweet sorghum bagasse (Cao et al. 2012), the method of dilute NaOH (2 %) autoclaving and H_2_O_2_ immersion caused removal of concentration of 91 % of lignin along with the 34 % of hemicellulose but retained cellulose with minimal loss.

**Table 1:** Chemical composition of native and pretreated lignocelluloses

Fraction	Percentage of dry weight of fractions in											
	Native				NaOH-treated				H_2_O_2_-treated			
	Sawdust	Wheat straw	Sugarcane bagasse	Rice bran	Sawdust	Wheat straw	Sugarcane bagasse	Rice bran	Sawdust	Wheat straw	Sugarcane bagasse	Rice bran
Hot water-soluble fraction	16.1	17.9	17.9	19.6	3.7	8.9	9.7	10.7	15.2	12.7	15.8	17.5
Hot ethanol-soluble fraction	6.9	10.7	11.1	9.8	2.8	6.7	7.1	11.4	10.1	9.3	7.9	8.6
Lignin	12.4	11.9	12.1	10.9	10.9	10.2	11.2	10.2	6.8	6.9	9.7	7.9
Hemicellulose	16.9	15.80	15.0	14.0	19.5	16.9	15.9	14.1	17.9	16.1	15.0	13.5
Cellulose	47.7	43.7	43.9	45.7	63.1	57.3	56.1	53.6	50.0	55.0	51.6	52.5

Values in the table are means of results of three experiments

### Optimization of saccharification of sawdust

**Fig. 1:**
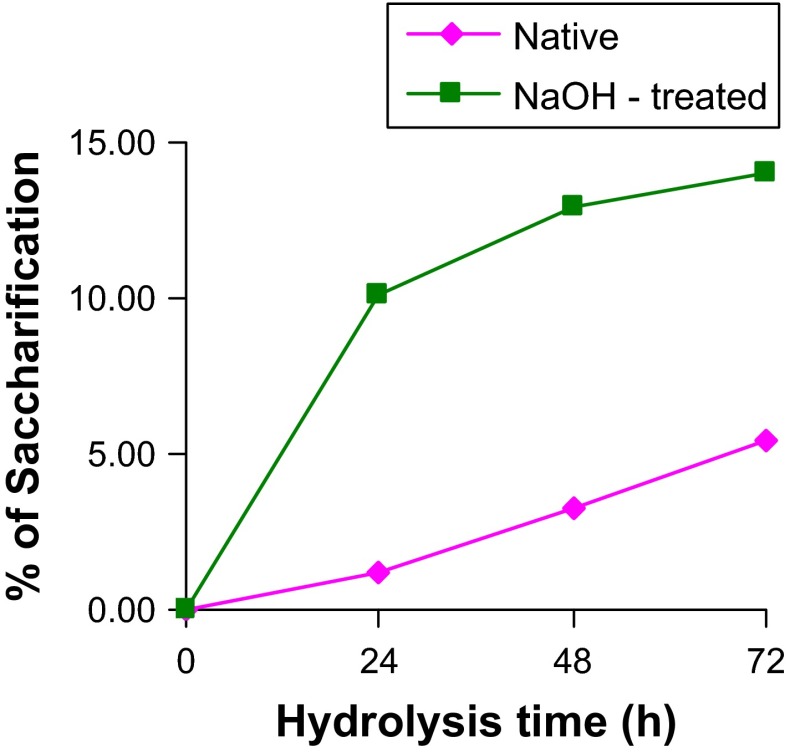
Saccharification of native and NaOH-treated sawdust by crude cellulase of *Aspergillus niger*

Experiments on saccharification in enzymatic digestion were conducted only on sawdust because of its high cellulose component. Yields of sugars in saccharification process upon incubation of alkali-treated and native sawdust with crude cellulase enzyme at loading of 7.56 FPU/g, 8.60 U/g of CMCase and 0.36 U/g β-glucosidase were compared (Fig. 1). Incubation of both treated and native sawdust with crude cellulase of *A. niger* resulted in release of soluble sugars in saccharification process. Higher saccharification efficiency (14 %) was recorded with alkali-treated saw dust as against 5.4 % with native sawdust. About threefold increase in hydrolysis of treated sawdust was observed in comparison to untreated sawdust. Similarly, about 47 % more glucose yields were obtained from enzymatic (25 FPU/g, 40 IU β-glucosidase/g) hydrolysis of 5 % rice straw pretreated with organosolve method in comparison to yields from untreated rice straw (Amiri et al. 2014). Incubation of crude cellulolytic-ligninolytic enzymatic extracts of *Pleurotus ostreatus* and *P. chrysosporium* with banana waste after sequential pretreatment with 3 % HCl first and then 3 % NaOH released reducing sugars up to 63.65 gL^−1^ (Xenia et al. 2011). In a majority of studies, cellulase enzyme of commercial grade was used at rate of loading higher than that used in the present study. For instance, saccharification of pretreated rice straw with 50 FPU of cellulase and 10 U of β-glucosidase gave yields (26.3 g/L) of reducing sugars higher than that (17.79 g/L) of pretreated sugar cane bagasse with the same enzyme load (Sukumaran et al. 2009). Pretreated rice straw (Remli et al. 2014) and pretreated wheat straw (Feng et al. 2014) were digested with cellulase at loading of 10–15 FPU/g with/without β-glucosidase. Saccharification of feedstocks with/without pretreatment was carried out with cellulase at loading rate of 30–60 FPU/g for solubilisation of sugars (Thomsen et al. 2012; Wang 2014; Zhang et al. 2012). Recovery of glucose increased with increase in dosage of cellulase from 5 U to 10 U/g along with 20 U of β-glucosidase (Li et al. 2014).

### Effect of temperature

**Fig. 2:**
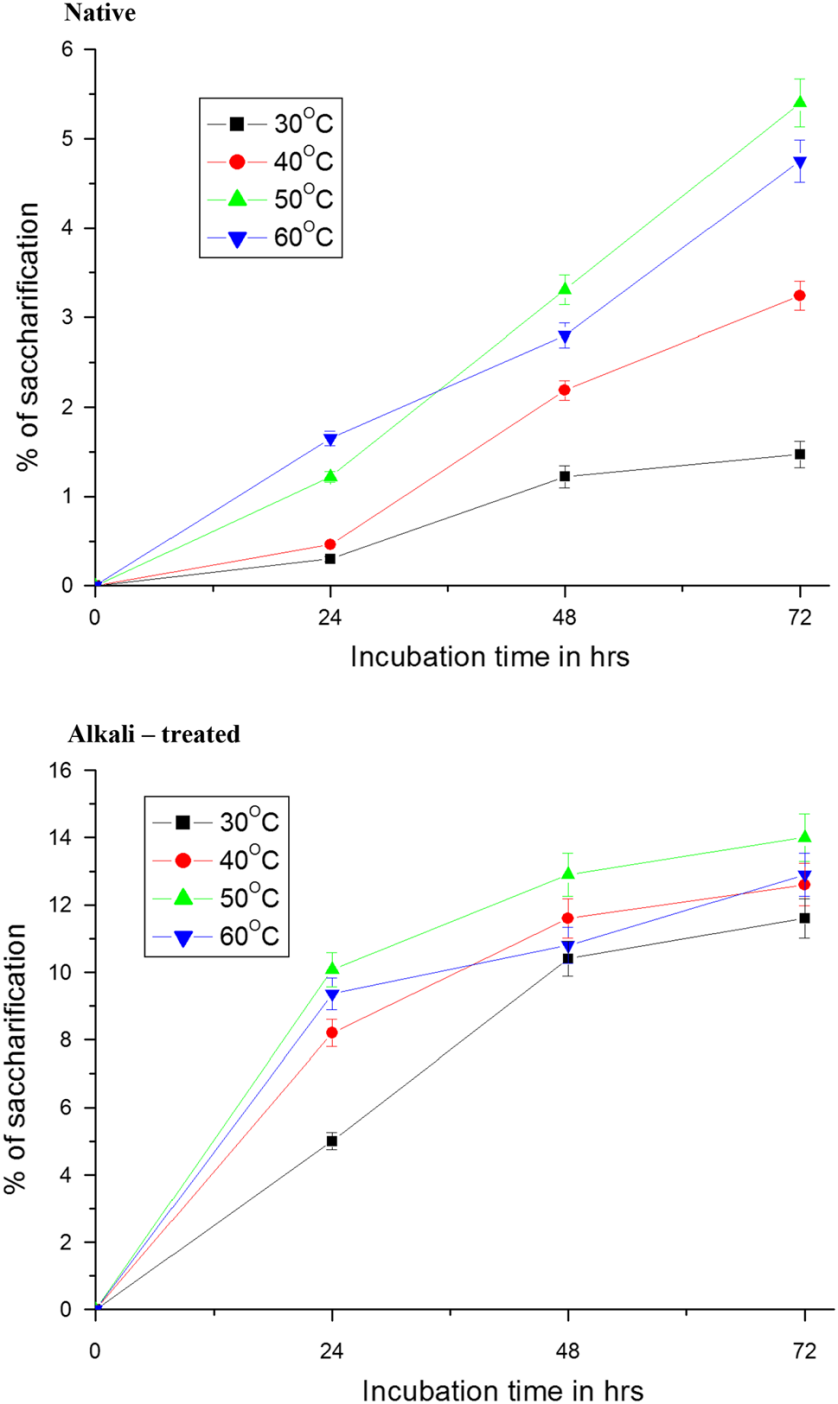
Effect of temperature on enzymatic hydrolysis of native and NaOH-treated sawdust by crude cellulase of *Aspergillus niger*

In order to increase the sugar production during the saccharification of pretreated sawdust, the main experimental variables—pH, temperature and substrate concentration–were further optimized in our final experiments. Saccharification of sawdust when carried out at 50 °C temperature caused maximum release of sugars (Fig. 2). After 72 h of incubation, approximately 5.4 and 14 % of substrate were hydrolysed at 50 °C temperature from native sawdust and treated sawdust, respectively. Temperatures above or below optimal temperature caused a significant decrease in the hydrolysis rate. These results clearly show that the optimal temperature for hydrolysis of native and alkali-treated sawdust was 50 °C. When the saccharification process of lignocelluloses proceeds at low temperature it would be desirable for industrial application because of less input of energy. The rate of hydrolysis was increased with increase in incubation time probably due to the attack of enzymes on amorphous regions in the initial stages (Desai et al. 1997). As the reaction proceeded and the amorphous regions were exhausted, the overall rate of hydrolysis rate slowed down due to slow attack of enzyme on crystalline regions. In addition to crystallinity, perhaps reactive cellulose chain ends were made during pretreatments and were hydrolysed by cellobiohydrolases much faster than the endoglucanase enzymes could replace them in the initial stages of hydrolysis (Converse 1993). In the same way, the optimum temperature for saccharification was found to be 50 °C in studies of Ortega et al. (2000). Crude extract of *A. niger* hydrolysed 80.3 % of alkali-treated wheat straw, the ammonium sulphate precipitated enzyme hydrolysed 79.1 % of substrate while dried moldy substrate caused 82.8 % hydrolysis at 50 °C after 48 h (Fadel 2000). Rice straw with/without pretreatment was subjected to enzymatic hydrolysis at 45 °C (Amiri et al. 2014). A maximum of 440 mg glucose/g dry biomass was obtained in enzymatic saccharification from *Erica* spp. pretreated at 180 °C with 2.75 % of sulfuric acid for 75 min in the presence of 0.25 g/g dry biomass of PEG 4000 (Gil et al. 2010).

### Effect of pH

**Fig. 3:**
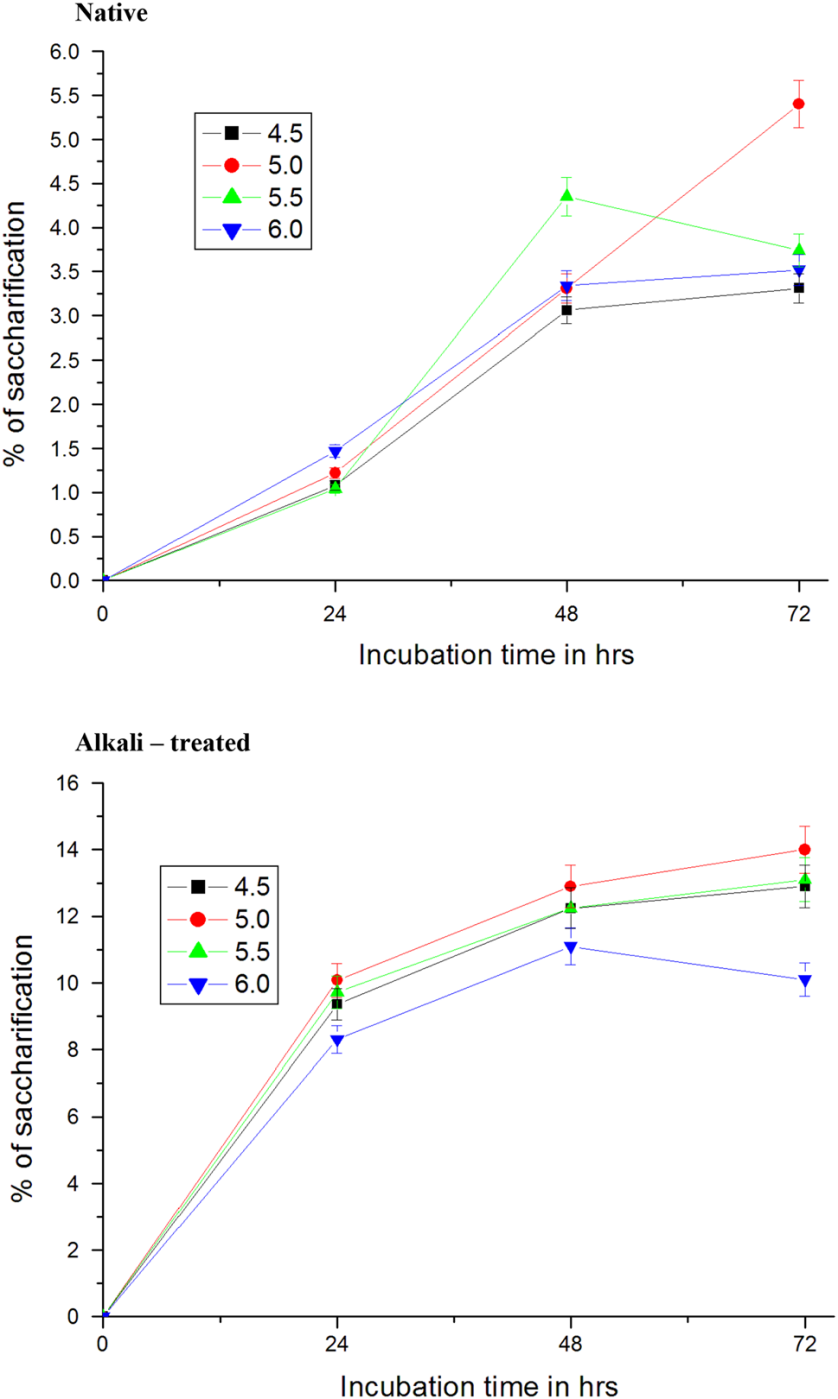
Effect of pH on enzymatic hydrolysis of native and NaOH-treated sawdust by crude cellulase of *Aspergillus niger*

Hydrolysis of alkali—treated sawdust with crude cellulase of *A. niger* at different pH was carried out and hydrolysis rates were measured. Figure 3 explains the influence of pH on saccharification of treated sawdust. Hydrolysis of treated sawdust after 72 h occurred at the maximal rate of 14 % at pH 5.0. Hydrolysis of sawdust at other pHs, 4.5, 5.5 and 6.0, was lower side in comparison to that of pH 5.0. Thus it is evident from these results that pH 5.0 was optimal for achieving maximum hydrolysis of sawdust with cellulase of *A. niger* Similarly, the optimum pH for saccharification of treated wheat straw was found to be 5.0 according to studies of Ortega et al. (2000). Crude extract of *A. niger* hydrolysed 80.3 % of alkali-treated wheat straw at pH 4.5 (Fadel 2000). The degradation of cell wall structure materials by cellulases derived from different organisms at different pH and temperature proceeded at different rates because the susceptibility of cellulosic substrates to enzymatic hydrolysis was dependent on a number of factors including structural features related to cellulose crystallinity (Fan et al. 1991; Mads et al. 2011), degree of polymerization (Sinitsyn et al. 1991), lignin content (Gharpuray et al. 1983) or surface area exposed to cellulases (Thompson et al. 1992).

### Effect of substrate concentration

**Fig. 4:**
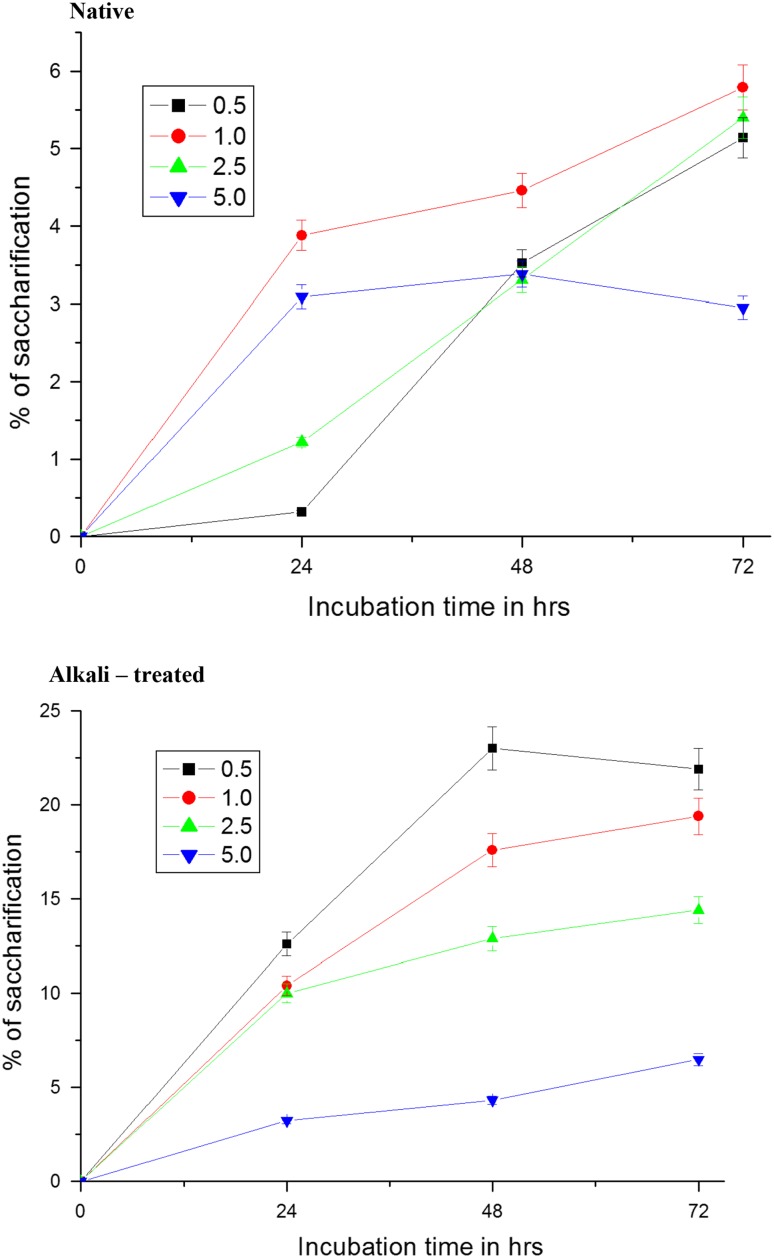
Effect of substrate concentration on enzymatic hydrolysis of native and NaOH-treated sawdust by crude cellulase of *Aspergillus niger*

Enzymatic hydrolysis of pretreated sawdust as function of substrate concentration is shown in Fig. 4. Saccharification on the pretreated sawdust at the lowest 0.5 % concentration occurred at maximal rate in 48 h, whereas saccharification on the native sawdust at the same concentration proceeded slowly. Twenty-three per cent of hydrolysis took place on treated sawdust as against 4 % on the native sawdust during 48 h. Increasing the sawdust concentration from 0.5 to 5 % decreased the saccharification rate. It is clear from the results of the present study that the pretreated sawdust at 0.5 % concentration was optimal for enzymatic hydrolysis with maximum yield of fermentable sugars at enzyme load used in the present study. Results on hydrolysis of alkali-treated sawdust after 48 h by *T. reesei* cellulase and *A. niger* β-glucosidase (Desai et al. 1997) were in agreement with these results of the present study. No improvement in yields of glucose in enzymatic hydrolysis with increase in loading of rice straw from 5 to 8 % (Amiri et al. 2014) was also in conformity with the results of the present study. The rate (6–14 %) of enzymatic saccharification of pretreated lignocellulose with locally produced cellulase (crude) was lower than the rate (33–63 %) of saccharification of same lignocellulose with commercial cellulase enzyme under similar conditions (Irfan et al. 2011). Concentration of cellulase enzyme used in this study for saccharification may not be adequate to support saccharification of pretreated sawdust at higher concentrations, but needs to be tested at different higher loading of cellulose enzyme. The association of cellulose with lignin and hemicellulose in the lignocellulosic materials is an important factor limiting the susceptibility to hydrolysis (Yang et al. 2011a). Higher rate of saccharification of pretreated sawdust with crude cellulase in the present study could be due to enhancement in the accessibility of cellulase to cellulose because of opening of cell wall structure with removal of the matrix. Loading of crude/pure cellulase in supplementation with high β-glucosidase at high concentrations may further improve the yields of the saccharification. For each pair, the mean difference, its standard deviation and the results of the *t* test were studied (Table 2). Basing on the *p* value of the *t* test, the differences in all pairs are significant. In all the conditions (Temp, pH, Substrate Conc.), the amount of glucose liberated was significantly different between native and alkali treated sawdust.

**Table 2:** Stastical analysis of the results

Paired samples test									
Pair		Paired differences 95 % Confidence Interval of the Difference					*t*	df	Sig. (2-tailed)
		Mean	Std. Deviation	Std. Error Mean	Lower	Upper			
1	pH Native—alkali treated	−8.43417	1.00491	0.29009	−9.07266	−7.79568	−29.074	11	0.000
2	Substrate conc. native—alkali treated	−9.48167	6.00127	1.73242	−13.29469	−5.66864	−5.473	11	0.000
3	Temperature native—alkali treated	−8.45250	1.41427	0.40827	−9.35109	−7.55391	−20.703	11	0.000

## Conclusions

As cellulose is encased in a matrix of lignin and hemicellulose in lignocelluloses, cellulose component is less inaccessible to enzyme hydrolysis. Of the two pretreatment methods—alkaline (NaOH) and peroxide (H_2_O_2_—treatment), tested on lignocelluloses, alkaline treatment had retained more cellulose component in all four lignocelluloses used in this study. Enzymatic saccharification of alkali-treated sawdust with highest cellulose content released higher yields of soluble sugars in comparison to native sawdust. Parameters—pH, temperature and substrate concentration affecting hydrolysis of sawdust—were optimized for increasing the rate of saccharification process. Hydrolysis of NaOH-treated sawdust with crude cellulase of A. *niger* yielded reducing sugars of 23 % under optimal conditions of 0.5 % substrate concentration, pH 5.0 and 50 °C temperature in 48 h in comparison to 4 % from native sawdust. It is clear from this study that pretreated lignocellulosic substrates can be tapped as a source for the production of fermentative sugars that can be subsequently used for different value addition products.
